# Description and Molecular Characterization of *Criconemoides iraqicus* n. sp. (Rhabditida: Criconematidae) From Iraq

**DOI:** 10.2478/jofnem-2025-0046

**Published:** 2025-10-05

**Authors:** Ahmed Malik Jumaah, Sedighe Azimi

**Affiliations:** Department of Plant Protection, Faculty of Agriculture, Shahid Chamran University of Ahvaz, Ahvaz, Iran

**Keywords:** D2–D3 expansion segments of LSU rDNA, ITS rDNA, Misan province, morphology, morphometric data, phylogeny, SSU

## Abstract

*Criconemoides iraqicus* n. sp. recovered from the rhizospheric soil of pomegranate in Misan province, Iraq, is described based on morphological and molecular data. The new species is characterized by its lip region comprised of two annuli, true submedian lobes absent, pseudolips present, body annuli smooth and with few anastomoses (*R* = 76–79), stylet 64.6–75.3 μm long, with anchor-shaped basal knobs, excretory pore at one to three annuli posterior to the pharynx base, vulva closed, vulval lips not projecting above body contour, and tail conical with one to three terminal lobes. Based on the number of body annuli, stylet length, smooth annuli, and shape of postvulval body region, *C*. *iraqicus* n. sp. is closely similar to *C. amorphus*, *C. ananasi*, *C. geraerti*, *C. informis*, *C. neoinformis*, and *C. tenuiannulatus*. The phylogenetic relationships of the new species with representatives of the family Criconematidae were reconstructed and discussed using partial sequences of the small subunit, D2–D3 expansion segments of the large subunit, and internal transcribed spacer regions of ribosomal DNA (SSU, LSU D2–D3, and ITS rDNA) based on Bayesian inference (BI). In phylogenetic trees, sequences of the new species formed clades with corresponding sequences of *C. geraerti, C. informis, Discocriconemella parasinensis,* and *D. sinensis* with different levels of relatedness.

The genus *Criconemoides* Taylor (1936) belongs to the family Criconematidae Taylor, 1936 (Thorne, 1949), which is distributed worldwide (Geraert, 2010). They are obligate ectoparasites on various plants and inhabit mostly sandy soils (Dropkin, 1955). Several species of the genus damage the roots of many economically important crops. It is essential to accurately identify these nematodes in order to manage them and to develop germplasm resistant to these pests (Cordero et al., 2012; Ferris et al., 2012).

In *Criconemoides*, true submedian lobes are absent, but six pseudolips are developed as outgrowths of the first annulus. The lateral ones are often reduced to low connections between the submedian ones, which superficially resemble true submedian lobes in lateral view. Compared with *Criconemoides*, *Mesocriconema* Andrássy (1965) has submedian lobes and open vulva (Brzeski et al., 2002; Geraert, 2010).

According to Geraert (2010), *Criconemoides* comprises 45 valid species. Recently, five other species were described from China (Maria et al., 2020) and Iran (Hosseinvand et al., 2022). Most species of the genus *Criconemoides* have been traditionally identified and described. Molecular techniques and electron microscope images significantly aid in validating taxonomic status and inferring phylogenetic relationships with other relevant species and genera (Cordero et al., 2012; Maria et al., 2020). Cryptic speciation has been documented in the case of criconematids using integrative taxonomic analyses; therefore, these data enhanced the hypothesis that criconematid nematodes are a hyper-diverse group of organisms (Olson et al., 2017; Maria et al., 2020; Clavero-Camacho et al., 2022).

There is very little information about plant-parasitic nematodes of the family Criconematidae in Iraq. *Mesocriconema antipolitanum* (De Guiran, 1963) Loof and De Grisse, 1989, *Criconemoides amorphus* De Grisse, 1967, *Hemicriconemoides chitwoodi* Esser (1960), *H. cocophilus* (Loos, 1949) Chitwood and Birchfield, 1957, *H. mangiferae* Siddiqi, 1961 have been reported from vineyard soils in Iraq, but morphological and morphometric data were not provided for the aforementioned species (Stephan et al., 1985). Recently, *Criconemoides informis* (Micoletzky, 1922) Taylor, 1936 has been reported from southern Iraq (Jumaah, 2024).

In this paper, an unknown species of the genus *Criconemoides* was recovered from Iraq. The present study aims to provide morphological, morphometric, and molecular data for the recovered new species and to determine its molecular phylogenetic affinities with other species of *Criconemoides* and other taxa in the family Criconematidae using three markers.

## Materials and Methods

### Nematode extraction and morphological observations

Several soil samples were collected from the rhizosphere of pomegranate (*Punica granatum* L.) in Misan province, Iraq. The centrifugal-flotation technique (Jenkins, 1964) was used to extract the nematodes from soil samples. The collected specimens were killed in a hot 4% formaldehyde solution and transferred to anhydrous glycerin, according to De Grisse (1969). Observations and measurements were conducted using a Leitz SMLUX light microscope (Leitz Corporation, Wetzlar, Germany) with a drawing tube. Some specimens were photographed using an Olympus BX51 light microscope (Olympus Corporation, Tokyo, Japan) with a Tucsen Michrome 20 digital camera.

### DNA extraction, PCR, and sequencing

For molecular analyses, single female specimens were picked out, examined in a drop of distilled water on a temporary slide under a light microscope, and transferred to 5 μl of TE buffer (10 mM Tris-Cl, 0.5 mM EDTA; pH 9.0) on a clean slide, then crushed using a cover slip. The suspension was collected by adding 10 μl TE buffer. The DNA samples were stored at −20°C until used as polymerase chain reaction (PCR) template. Primers for amplification of 18S rDNA were the forward primer SSU22F (5′-TCCAAGGAAGGCAGCAGGC-3′) and reverse primer SSU13R (5′-GGGCATCACAGACCTGTTA-3′) (Dorris et al., 2002). Primers for 28S rDNA D2–D3 amplification were forward primer D2Ab (5′-ACAA GTACCGTGAGGGAAAGT-3′) and reverse primer D3B (5′-TCGGAAGGAACCAGCTACTA-3′) (De Ley et al., 1999). Primers for amplification of ITS rDNA were forward primer rDNA1 (5′-TTGATTACGTCCCTGCCCTTT-3′) and reverse primer rDNA1.58S (5′-ACGAGCCGAG TGATCCACCG-3′) (Subbotin et al., 2000). To amplify the above-mentioned DNA fragments, the PCR was performed as described by Azimi and Abdolkhani (2023). Amplification success was evaluated by electrophoresis on 1% agarose gel. The PCR products were sequenced using an Applied Biosystems 3500 (ABI) sequencer, Pishgam Corporation, Tehran, Iran. The newly obtained sequences of the new species were deposited into the GenBank database (accession numbers PV603370 for 18S rDNA, PV603399, PV603400 for LSU rDNA D2-D3, and PV604659, PV604660 for ITS rDNA).

### Phylogenetic analyses

The newly obtained sequences of the SSU rDNA, D2–D3 fragments of LSU rDNA, and ITS rDNA were compared with those of other nematode species available in the GenBank database using the BLAST homology search program, and the selected sequences to reconstruct each phylogeny were retrieved. The sequences were aligned with Clustal X version 2 using the default parameters (Larkin et al., 2007). The three alignments were edited manually in the MEGA7 program (Kumar et al., 2016). The base substitution model was selected using MrModeltest 2 (Nylander, 2004) based on the Akaike information criterion. A general time-reversible model, including among-site rate heterogeneity and estimates of invariant sites (GTR + G + I), was used in all three phylogenies. Sequences of the genus *Paratylenchus* Micoletzky, 1922, were chosen as outgroup taxa according to the previous studies (Jahanshahi Afshar et al., 2019; Maria et al., 2020). The sequences with accession numbers AY284631 and AY284633 were used as outgroups in SSU phylogeny; KF242218 and KF242228 were used as outgroups in LSU rDNA phylogeny, and KF242271 and KF242247 were used as outgroups in ITS phylogeny.

Bayesian analysis was performed to infer the phylogenetic trees using MrBayes v3.1.2 (Ronquist and Huelsenbeck, 2003), running the chains for 4 million generations. After discarding burn-in samples and evaluating convergence, the remaining samples were retained for further analyses. The Markov chain Monte Carlo (MCMC) method within the Bayesian framework was used to determine equilibrium distribution and help estimate the posterior probabilities of the phylogenetic trees (Larget and Simon, 1999) using the 50% majority rule. Bayesian posterior probability (BPP) values higher than 0.50 are shown on the corresponding clades. The output files of the phylogenetic program were visualized using Dendroscope v3.2.8 (Huson and Scornavacca, 2012) and trees were digitally drawn in CorelDRAW software version 23 (Corel Corporation, Ottawa, Canada).

## Results

### Taxonomy

**Figure 1:**
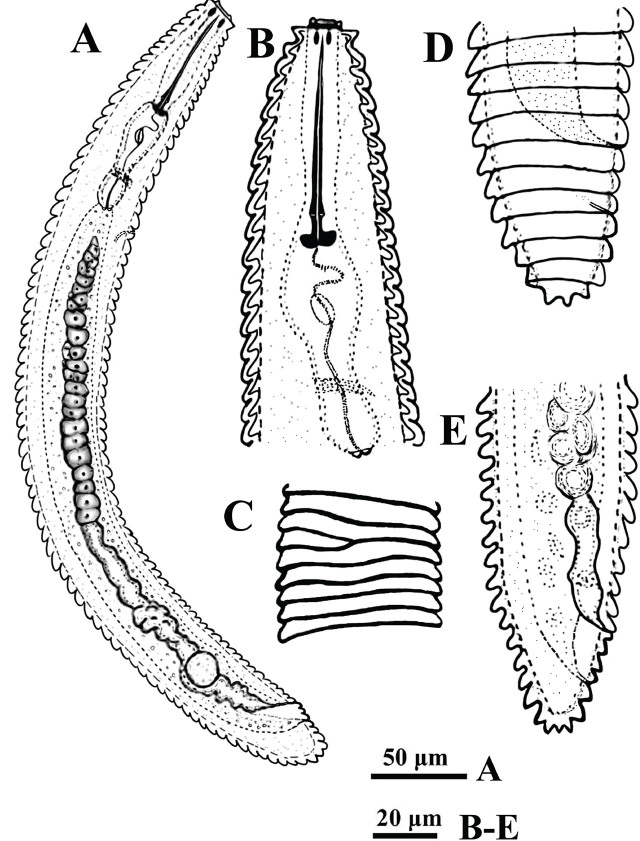
Line drawings of *Criconemoides iraqicus* n. sp. from Iraq. Female. (A) Entire body; (B) Anterior body region; (C) Cuticular annuli showing anastomosis; (D,E) Posterior body region.

**Figure 2:**
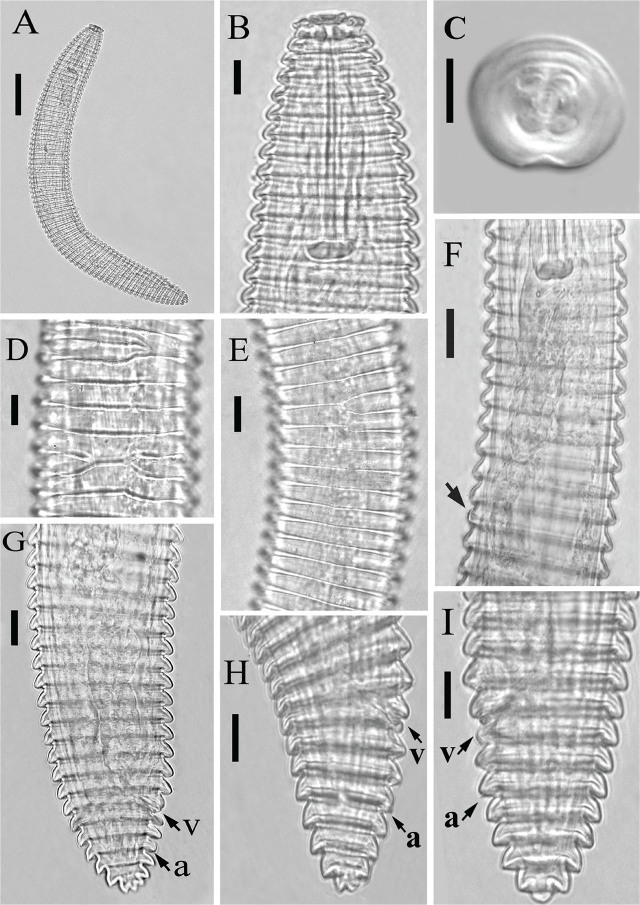
Light photomicrographs of *Criconemoides iraqicus* n. sp. from Iraq. Female. (A) Entire body; (B) Anterior body region; (C) *En face* view; (D,E) Cuticular annuli showing anastomosis; (F) Pharyngeal region (the arrow indicates the excretory pore); (G–I) Posterior body region. Arrowheads showing position of vulva (v) and anus (a). Scale bars: A = 50 μm; B–I = 10 μm.

*Criconemoides iraqicus* n. sp. (Figs. 1 and 2; Table 1).

**Table 1:** Morphometrics of *Criconemoides iraqicus* n. sp. from Misan province, Iraq.

**Character**	**Holotype Female**	**Paratypes**	

		**Females**	**Juveniles**
n	1	5	6
L	455	474.2 ± 21.8 (443–496)	292.3 ± 26.5 (278–321)
a	10.3	10.3 ± 0.9 (9.4–11.6)	13.1 ± 0.3 (12.4–13.6)
b	3.5	3.8 ± 0.2 (3.5–4.2)	3.5 ± 0.3 (3.3–4.1)
c	25.3	29.3 ± 2.7 (24.9–33.3)	23.4 ± 1.6 (21.3–25.2)
c′	0.8	0.7 ± 0.1 (0.6–0.8)	0.8 ± 0.2 (0.7–0.9)
o	8.6	8.4 ± 0.3 (7.8–8.9)	6.8 ± 0.2 (6.3–7.2)
DGO	6.5	6.1 ± 0.4 (5.3–6.8)	3.7 ± 0.4 (3.1–4.3)
V	92.3	91.5 ± 0.3 (91.2–92.5)	–
Stylet length	75.3	68.2 ± 3.6 (64.6–75.3)	47.4 ± 0.5 (46.6–50.3)
m	79.7	81.9 ± 2.4 (78.6–85.3)	78.2 ± 2.6 (73.2–81.4)
Stylet Knob height	4.5	4.6 ± 0.3 (4.2–5.3)	3.7 ± 0.3 (2.8–4.1)
Stylet Knob width	12	10.8 ± 0.5 (10.2–12.2)	8.9 ± 0.3 (7.8–10.2)
Anterior end to excretory pore	134	132.4 ± 2.8 (129–137)	90.6 ± 1.2 (87–95)
Body width	44.2	45.2 ± 2.2 (43.2–47.6)	25.5 ± 1.6 (23.2–28.7)
Anal body width	23	22.3 ± 1.2 (20.5–23.8)	14.1 ± 0.9 (12.8–15.4)
Vulval body width	32	30.7 ± 1.6 (27.7–33.8)	–
Lip region-vulva	420	442.3 ± 27.8 (396–470)	–
Vulva-tail terminus	30	34.8 ± 1.2 (29.4–38.9)	–
First lip annulus diam.	11.5	11.4 ± 0.9 (10.0–12.9)	10.4 ± 0.6 (9.6–11.8)
Second lip annulus diam.	15.2	13.2 ± 1.1 (11.6–15.7)	12.7 ± 1.2 (10.4–14.6)
First body annulus diam.	18	16.7 ± 1.2 (14.3–18.5)	15.3 ± 1.3 (13.5–17.2)
Second body annulus diam.	21.5	20.6 ± 2.3 (17.5–22.8)	18.8 ± 1.5 (16.4–20.1)
Pharynx length	128	121.8 ± 4.1 (117–130)	89.2 ± 3.2 (87–93)
Annulus width	6.8	8.1 ± 0.3 (6.3–8.7)	3.2 ± 0.1 (2.8–3.6)
Tail length	18	16.8 ± 1.5 (14.3–19.1)	16.5 ± 1.2 (13.4–18.9)
R	79	77.8 ± 2.2 (76–79)	82.7 ± 2.1 (77–84)
RSt	14	13.2 ± 0.8 (12–15)	12.2 ± 0.8 (11–13)
ROes	21	19.5 ± 1.4 (20–24)	23.5 ± 1.9 (20–25)
Rex	23	21.6 ± 1.3 (22–25)	25.2 ± 2.1 (22–27)
RV	7	8.4 ± 0.6 (7–9)	–
RVan	3	2.5 ± 0.5 (2–3)	–
Ran	4	3.6 ± 0.4 (3–4)	3.9 ± 0.5 (3–5)
VL/VB	0.9	1.0 ± 0.1 (0.9–1.3)	–
St%L	16.5	16.1 ± 0.3 (14.2–17.2)	15.7 ± 0.4 (15.4–16.6)

All measurements are in μm and in the form: mean ± SD (range).

DGO, Dorsal gland orifice.

### Description

#### Female

Body ventrally arcuate following heat relaxation. Body annuli smooth. Anastomoses three to four at the anterior half. Lip region with two annuli, the first labial annulus separated from the second by a narrow constriction, slightly directed forwardly. *En face* view of the lip region shows oral disc is rounded, amphidial openings are oval-shaped, true submedian lobes absent, six pseudolips present, lateral ones reduced, the subdorsal and subventral ones enlarged, appearing as outgrowths on the first annulus in lateral view, resembling submedian lobes (Fig. 2C). Stylet robust with anchor-shaped basal knobs. The dorsal gland orifice (DGO) is at a relatively short distance from the stylet base. Pharynx criconematoid. Nerve ring encircling the isthmus. The secretory-excretory pore is one to three annuli posterior to the pharynx base. Reproductive system monodelphic-prodelphic, outstretched, spermatheca round to oval, empty in most specimens, with few rounded sperm in one specimen, vulva closed, vulval lips not projecting above the body contour, anterior and posterior annuli surrounding the vulva larger than the preceding body annuli. Anus hardly visible, two to three annuli posterior to the vulva. Tail conical with one to three terminal lobes.

#### Male

Not found.

#### Juvenile

**Figure 3:**
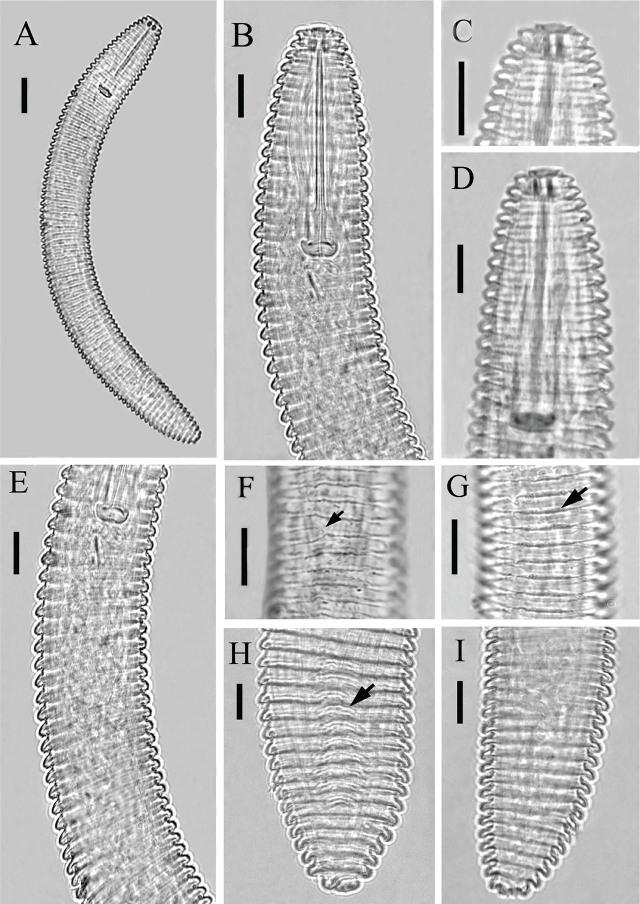
Light photomicrographs of *Criconemoides iraqicus* n. sp. from Iraq. Juveniles. (A) Entire body; (B–D) Anterior body region; (E) Pharyngeal region; (F–H) Cuticular annuli showing anastomoses and ornamentation; (I) Posterior body region. Scale bars: A = 20 μm; B–I = 10 μm.

The recovered juveniles look similar to females except for their smaller bodies and undeveloped sexual organs. Annuli retrorse with smooth to rough margin. Outgrowths resembling submedian lobes are present at the first labial annulus. Anastomoses two to three from the middle to near the end of the body (Fig. 3).

### Type host and locality

This population was recovered from the rhizospheric soil of pomegranate collected from Al-Kahla city in Misan province, Iraq. The GPS information of the sampling site is 31°40′53.29″N, 47°16′44.14″E.

### Etymology

The specific epithet refers to the country name where it was found.

### Type material

The holotype female, two paratype females, and four paratype juveniles were deposited in the nematology laboratory of the Department of Plant Protection, Shahid Chamran University of Ahvaz, Ahvaz, Iran. Two paratype females and two juveniles were also deposited at the Wageningen Nematode Collection (WaNeCo), Wageningen, The Netherlands. The Life Science Identifier code (LSID) for this publication is:http://zoobank.org/urn:lsid:zoobank.org:pub:4915A3B8-92CE-4D46-AC26-09F7FA496724 .

### Diagnosis and relationships

*Criconemoides iraqicus* n. sp. is mainly characterized by a lip region comprised of two annuli, a rounded oral disc, true submedian lobes absent, six pseudolips present, lateral ones reduced, the subdorsal and subventral ones enlarged, appearing as outgrowths resembling submedian lobes on the first annulus in lateral view, body annuli smooth and with few anastomoses, a stylet with anchor-shaped basal knobs, an excretory pore at one to three annuli posterior to the pharynx base, vulva closed, vulval lips not projecting above body contour, and tail conical with one to three terminal lobes.

Based on the number of body annuli, stylet length, smooth annuli, and shape of postvulval body region, C. *iraqicus* n. sp. is closely similar to *C. amorphus*, *C. ananasi* Misra and Edward, 1972, *C. geraerti* Maria, Miao, Cai, Tian, Castillo & Zheng, 2020, *C. informis*, *C. neoinformis* Hosseinvand, Eskandari, Palomares-Rius, Castillo, Abolafia & Ghaderi, 2022, and *C. tenuiannulatus* (Tulaganov, 1949) Raski and Golden, 1966.

It differs from *C. amorphus* by having a higher number of body annuli (76–79 *vs* 55–76), a higher number of annuli from excretory pore to anterior end (22–25 *vs* 17–22), and a relatively lower VL/VB ratio (0.9–1.3 *vs* 1.1–1.7). From *C. ananasi*, by the higher number of body annuli (76–79 *vs* 58–75), anastomoses present (*vs* absent), the higher number of annuli from excretory pore to anterior end (22–25 *vs* 20), and position of the excretory pore (posterior to pharyngeal bulb base *vs* at the junction of pharynx and intestine or anterior to the pharyngeal bulb base). From *C. geraerti*, by longer stylet (64.6–75.3 μm *vs* 57.0–62.7 μm), longer pharynx (117–130 μm *vs* 100.0–109.8 μm), position of the excretory pore (posterior to pharyngeal bulb base *vs* at the same level, or one to two annuli anterior to pharyngeal bulb base), lower V ratio (91.2–92.5 *vs* 92.6–94.2) and longer distance from vulva to tail terminus (29.4–38.9 μm *vs* 19.4–27.4 μm). From *C. informis*, by the slightly higher number of body annuli (76–79 *vs* 48–77), elevated labial disc absent (*vs* present), and rounded lip annuli (*vs* usually directed sideways). From *C. neoinformis*, by the shorter body (443–496 μm *vs* 522–585 μm), the higher number of body annuli (76–79 *vs* 57–63), shorter stylet (64.6–75.3 μm *vs* 75.5–88.0 μm), anastomoses present (*vs* absent), higher number of annuli from excretory pore to anterior end (22–25 *vs* 18–22), shorter distance of anterior end to excretory pore and pharynx length (129–137 *vs* 143–189 and 117–130 μm *vs* 133–150 μm, respectively), and shorter tail (14.3–19.1 μm *vs* 20–50 μm). From *C. tenuiannulatus*, by shorter body (443–496 μm *vs* 600 μm), fewer body annuli (76–79 *vs* 84–86), lower RV ratio (7–9 *vs* 6), higher RVan ratio (2–3 *vs* 1 and lower V ratio (91.2–92.5 *vs* 94).

### Molecular characterization and phylogenetic relationships

#### Partial SSU rDNA phylogeny

**Figure 4:**
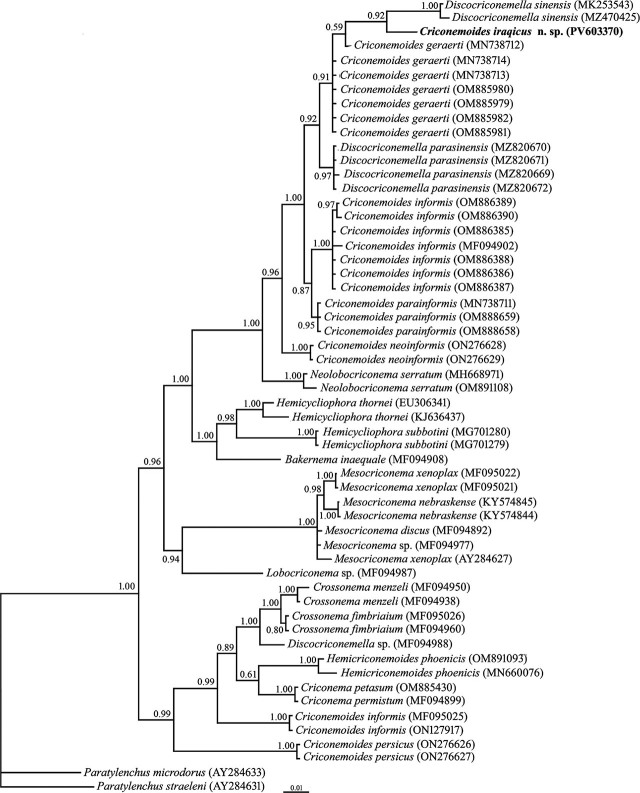
Bayesian 50% majority rule consensus tree inferred from analysis of the SSU rDNA sequence of *Criconemoides iraqicus* n. sp. under the GTR + G + I model. BPP values more than 0.50 are given for appropriate clades. New sequence is indicated in bold. BPP, Bayesian posterior probability.

To determine the phylogenetic relationships of *Criconemoides iraqicus* n. sp. with other nematode species using the SSU sequence, a newly obtained 991 nt long partial sequence of SSU rDNA with accession number PV603370 was used. The BLAST search using this sequence revealed it has 99.09% identity with SSU sequences of isolates of *C. geraerti* (OM885979-OM885982, MN738713-MN738714), *C. informis* (MF094902), and *Discocriconemella sinensis* Maria, Cai, Subbotin & Zheng, 2019 (MK253543, MZ470425). Sequence variation between the new and aforementioned sequences was nine mismatches. A total of 54 SSU sequences of the family Criconematidae were used for SSU phylogeny. This dataset comprised 1,747 total characters. The phylogenetic tree inferred using this dataset is presented in Figure 4. In this tree, the newly generated sequence of the new species has formed a clade with the corresponding sequences of *D. sinensis*. The clade containing these sequences is sister to the SSU sequence of *C. geraerti*.

#### D2–D3 fragment of LSU rDNA phylogeny

**Figure 5:**
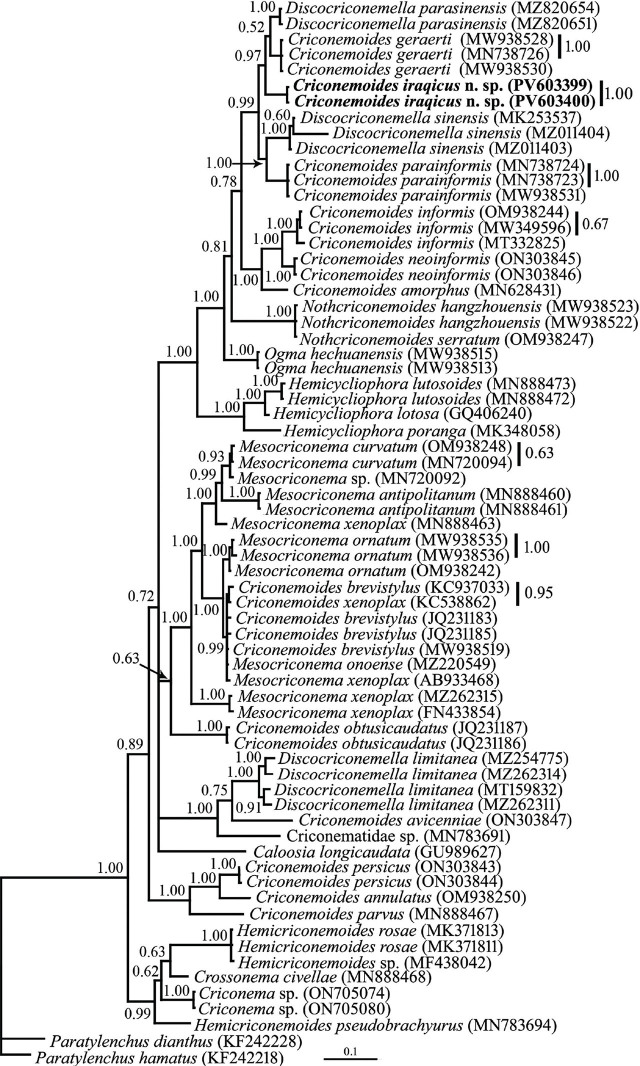
Bayesian 50% majority rule consensus tree inferred from analysis of the D2–D3 domains of the LSU rDNA sequences of *Criconemoides iraqicus* n. sp. under the GTR + G + I model. BPP values more than 0.50 are given for appropriate clades. New sequences are indicated in bold. BPP, Bayesian posterior probability.

To reconstruct the LSU rDNA tree, two identically aligned 719 nt long partial sequences of D2–D3 region with accession numbers PV603399 and PV603400 were used. The BLAST search using these sequences revealed they have 96.21% identity (yielded from 23 mismatches and 4 gaps) with the LSU sequence of *C. geraerti* (MN738727). A total of 66 LSU sequences of the family Criconematidae were used in the LSU phylogeny. This dataset comprised 783 total characters. The phylogenetic tree inferred using this dataset is presented in Figure 5. The newly generated LSU sequences of the new species have formed a clade with corresponding sequences of *C. geraerti* and *Discocriconemella parasinensis* Li, Munawar, Castillo & Zheng, 2022, with high support (BPP = 0.97).

#### Partial ITS rDNA phylogeny

**Figure 6:**
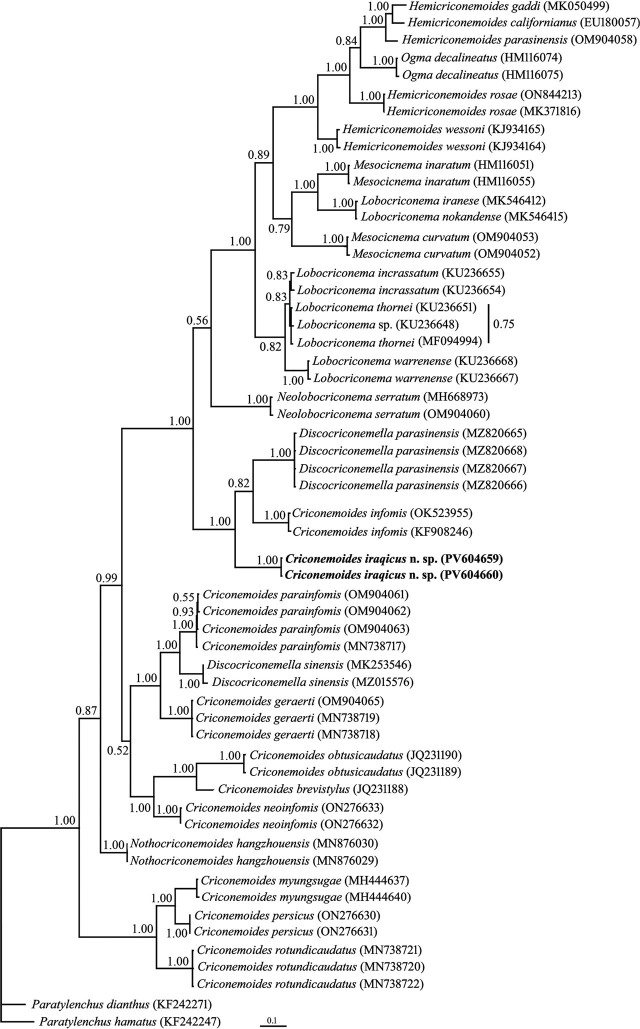
Bayesian 50% majority rule consensus tree inferred from analysis of the ITS rDNA sequence of *Criconemoides iraqicus* n. sp. under the GTR + G + I model. BPP values more than 0.50 are given for appropriate clades. New sequences are indicated in bold. BPP, Bayesian posterior probability.

Two identically aligned sequences of ITS rDNA, each 568 nt long (PV604659 and PV604660), were used in ITS phylogeny. A BLAST search using the ITS sequences of the new species revealed 89.30% identity with the corresponding locus of *D. parasinensis* (MZ820667-MZ820668). Sequence variations between these sequences consisted of 50 mismatches and 23 gaps. Fifty-six sequences of the family Criconematidae were included in the ITS phylogeny. The phylogenetic tree inferred using this dataset is presented in Figure 6. The ITS sequence of the new species formed a clade with those of *D. parasinensis* and *C. informis,* with maximal support.

## Discussion

The purpose of this study was to characterize a new Iraqi species of the genus *Criconemoides*. Identification of *Criconemoides* species based solely on morphology and morphometric is problematic and unreliable. As a result, an integrative method utilizing molecular and morphological data was employed to study the new species.

Ribosomal and mitochondrial markers are reliable tools for the accurate identification of *Criconemoides* spp., especially in the case of cryptic species found in this group (Maria et al., 2020; Hosseinvand et al., 2022). Several cryptic species have been reported in criconematids, and accordingly, these data support the hypothesis that criconematid nematodes are a hyperdiverse group of organisms (Maria et al., 2020; Clavero-Camacho et al., 2022).

A study on the phylogeny of Criconematoidea Taylor, 1936 based on 18S rDNA sequences revealed that *Criconemoides* is a paraphyletic taxon and suggested that key morphological characters used in the classification of Criconematidae are not homologous (Powers et al., 2017). The results of the present study using more sequences of more species showed it is polyphyletic based on three markers.

The present study represents the first integrative taxonomic study on Criconematidae in Iraq. Future surveys will further reveal the diversity of criconematids occurring in the country.
